# Investigation and Optimization of the Performance of an Air-Coil Sensor with a Differential Structure Suited to Helicopter TEM Exploration

**DOI:** 10.3390/s150923325

**Published:** 2015-09-15

**Authors:** Chen Chen, Fei Liu, Jun Lin, Yanzhang Wang

**Affiliations:** 1 College of Instrumentation & Electrical Engineering, Jilin University, Changchun 130061, China; E-Mails: cchen@jlu.edu.cn (C.C.); feiliu14@jlu.edu.cn (F.L.); lin_jun@jlu.edu.cn (J.L.); 2 Key Laboratory of Geo-exploration Instruments, Ministry of Education of China, Changchun 130061, China

**Keywords:** helicopter TEM exploration, ACS, common-mode noise suppression, specification optimization

## Abstract

An air-coil sensor (ACS) is a type of induction magnetometer used as a transducer to measure the variations of a magnetic field. This device is widely applied in helicopter transient electromagnetic method (TEM) exploration. Most helicopter TEM explorations generate common-mode noise and require extreme ACS specifications, both of which inevitably challenge geophysical explorations. This study proposes a differential air-core coil combined with a differential pre-amplifier to reduce the common-mode noise induced in exploration surveys. To satisfy the stringent performance requirements, including the geometric parameters and electrical specifications, the physical calculations in theory and the equivalent schematic of an ACS with noise location are investigated, respectively. The theory calculation and experimental result for the optimized ACS are then compared on the basis of a differential structure. Correspondingly, an ACS is constructed with a mass, resultant effective area, 3 dB bandwidth, signal-to-noise ratio, and normalized equivalent input noise of 2.5 kg, 5.5 m^2^ (diameter is 0.5 m), 71 kHz, 20 (the varying magnetic field strength is 1 nT/s), and 5.43 nV/m^2^, respectively. These data are superior to those of the traditional induction sensor 3D-3. Finally, a field experiment is performed with a fabricated sensor to show a valid measurement of the time-varying magnetic field of a helicopter TEM system based on the designed ACS.

## 1. Introduction

The helicopter transient electromagnetic method (TEM) is a popular geophysical method that is mainly used by three commercially operational companies: Aeroquest Ltd. (AeroTEM, Mississauga, ON, Canada), Geotech Ltd. (VTEM, Aurora, Canada), and THEM Geophysics Inc. (THEM, Gatineau, QC, Canada) [1]. An air-coil sensor (ACS), which is well known as the search coil magnetometer of a helicopter TEM system, is one of the most important sensors used to measure the variations of a magnetic field [2,3,4]. The ACS is widely used in space research, geophysical exploration, and other related studies because of its wide 3 dB bandwidth, low noise, and robustness [5,6]. In different cases, helicopter TEM exploration requires the physical parameters (e.g., mass, size), 3 dB bandwidth, and equivalent input noise (EIN) of an ACS in a different and strict manner [7]. For instance, the EIN of ACS for helicopter TEM exploration should be smaller than the spectra magnitude of the natural magnetic field to obtain efficient measurements. Furthermore, the 3 dB bandwidth should be higher than 50 kHz [8]. An ACS generally consists of two parts, namely an air-core coil and a pre-amplifier. An air-core coil is composed of a wound coil without a magnetic core and acts as a transducer that converts the rate of change of flux density into an output voltage signal derived from the fundamental Faraday’s law of induction. A pre-amplifier is an electronic amplifier used to amplify the possibly extremely weak signals in a wideband and plays an important role in signal-to-noise ratio (SNR) [9]. A pre-amplifier is considered the most important part of a helicopter TEM receiver because it directly affects performance. Thus, the custom design and fabrication of an ACS with optimized performance are necessary.

At present, the commercial ACSs for helicopter TEM exploration mainly include a rigid-coil sensor from Geonics Ltd. (PROTEM, Mississauga, ON, Canada) and a 3D-3 sensor [10], which have high-cut limits of 3 and 28 kHz and equivalent areas of 31.4 and 200 m^2^, respectively. Although the equivalent areas of such sensors satisfy the requirement of helicopter TEM, their bandwidth is narrow, thus limiting the detection depth in helicopter TEM exploration. The MTEM-AL sensor developed by Phoenix Geophysics (Canada) has an equivalent area of 100 m^2^, and its bandwidth can reach up to 50 kHz. Given such parameters, the sensor from Phoenix can satisfy the requirement of helicopter TEM exploration. However, its diameter is as large as 2.1 m, which cannot meet the ACS size requirement of a helicopter TEM system. Therefore, a custom ACS with optimized specifications for helicopter TEM exploration should be developed.

This manuscript (a) presents an optimized ACS combined with differential air-core coils and pre-amplifiers to reduce the common-mode noise; (b) investigates the physical calculation of air-core coil in theory and the electrical equivalent schematic with noise source to overcome the stringent performance requirements; (c) analyzes the technical specifications of an ACS, such as mass, size, bandwidth, EIN, and SNR; (d) presents optimization research for ACS specifications to meet the stringent requirements for helicopter TEM application; and (e) presents performance tests and field experiments on an ACS in comparison with an AeroTEM system to verify the valid measurement of the time-varying magnetic field of the optimized ACS.

The rest of the paper is organized as follows. Section 2 illustrates the physical structure and electrical equivalent model of the air-core coil. Section 3 presents the equivalent schematic of ACS with noise location. Section 4 describes the optimized performance research for ACS with a differential structure. To verify the performance of the designed ACS, shielding room measurements and field experiments are conducted to show the feasibility and reliability of the fabricated ACS. Finally, Section 5 provides the conclusion of the study and cites recommendations for future research.

## 2. Equivalent Electrical Model of an Air-Core Coil

**Figure 1:**
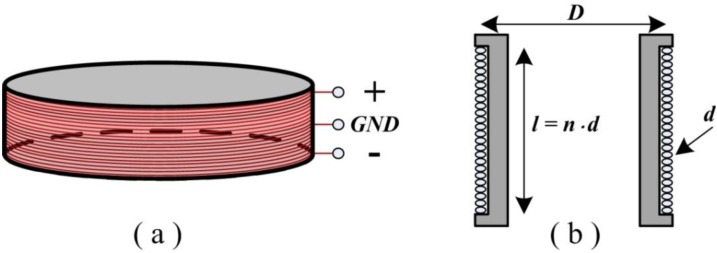
(**a**) Air-core coil with a differential structure; (**b**) typical design of an air-core coil (*l*—length of the air-core coil, *D*—diameter of the air-core coil, *d*—diameter of wire, and *n*—number of turns).

To reduce common-mode noise, the ACS uses two mirror air-core coils that are connected in a series and are wound by several turns of wire (Figure 1a) [3]. The connection of these air-core coils is used as a common ground connected to a differential pre-amplifier. As depicted in Figure 1b, a typical air-core coil is designed with a single layer [3] to decrease the distributed capacitance and increase the 3 dB bandwidth.

**Figure 2:**
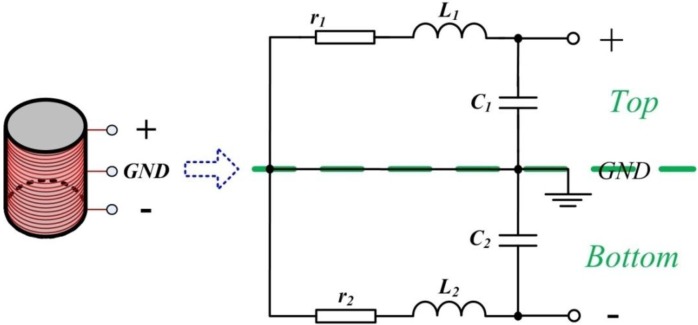
Schematic of the air-core coil, where *r*, *L*, and *C* are the resistance, inductance, and capacitance of the air-core coil, respectively.

The ACS coils are wound by several turns of wire, which may correspond to a series combination of resistor, inductor, and capacitor as a second-order circuit (Figure 2) [3].

Given the symmetrical structure of the air-core coil, two second-order circuits are constructed in parallel with the common ground. The specifications of the air-core coil (e.g., *D* and *n*) can directly affect the quality of the receiving signal, which emerges as a longitudinal resolution in helicopter TEM exploration.

To further analyze the mass, sensitivity, and SNR of the air-core coil, the area of an annulus of wood of the frame of a single-layer air-core coil is determined by using the following formula:

(1) $$A=\frac{\pi }{4}\cdot (D^{2}-{\left(D-0.1\right)}^{2})$$

Considering that many turns of wire are wound in a 0.1 m-thick ring wooden frame, the mass of the air-core coil is approximately calculated with wood density ρ.

(2) $$M=\rho \cdot \frac{\pi }{4}\cdot \left[(D^{2}-{\left(D-0.1\right)}^{2})\cdot n\cdot d+{\left(D-0.1\right)}^{2}\cdot 0.003\right]$$

where $\rho \cdot \frac{\pi }{4}\cdot {\left(D-0.1\right)}^{2}\cdot 0.003$ is the mass of the center wooden connection shown in Figure 10.

For the electrical performance analysis, the resolution of the air-core coil is limited by the self-thermal noise. This noise *V_t_* depends on the resistance *r* of the air-core coil and has a temperature *T* with a coefficient equal to the Boltzman factor *k_B_* = 1.38 × 10^−23^ W·s/K.

(3) $$V_{t}=\sqrt{4\cdot k_{B}\cdot T\cdot r}=\sqrt{4\cdot k_{B}\cdot T\cdot \rho_{r}\cdot \frac{n\cdot \pi \cdot D}{\pi \cdot {\left(\frac{d}{2}\right)}^{2}}}=\frac{4\sqrt{k_{B}\cdot T\cdot \rho_{r}\cdot n\cdot D}}{d}$$

where $\rho_{r}$ is the electrical resistivity of wire.

According to Faraday’s law of induction, the output signal *V* induced in a coil subjected to a varying magnetic field is given by the following equation:

(4) $$V=-n\cdot \frac{d\varphi }{dt}=-n\cdot A\cdot \frac{dB}{dt}=-n\cdot \frac{\pi }{4}\cdot D^{2}\cdot \frac{dB}{dt}$$

where *Φ* is the magnetic flux passing through the air-core coil, and $\frac{dB}{dt}$ is the magnitude of the varying magnetic field. The SNR of the air-core coil is expressed as

(5) $$SNR=-\frac{\pi }{16}\cdot \frac{d}{\sqrt{k_{B}\cdot T\cdot \rho_{r}}}\cdot \sqrt{n}\cdot D^{\frac{3}{2}}\cdot \frac{dB}{dt}$$

The equations cited in the preceding paragraphs indicate that parameters *n* and *D*, as the stringent physical requirements for the mass and size of the ACS, are limited by the load and dimension capacity in helicopter TEM exploration. For the electrical performance, the output signal *V* increases proportionally to *nD^2^*, and the self-thermal noise *V_t_* only goes up by $\sqrt{nD}$. Therefore, the optimum values of *D* and *n* for an air-core coil can be determined by considering the required SNR. The discussed parameters of the air-core coil determine the electrical equivalent parameters (*i.e.*, resistance, inductance, and capacitance), which are critical to the selection of the operational amplifier for a differential pre-amplifier.

## 3. Equivalent Schematic of ACS with Noise Source

**Figure 3:**
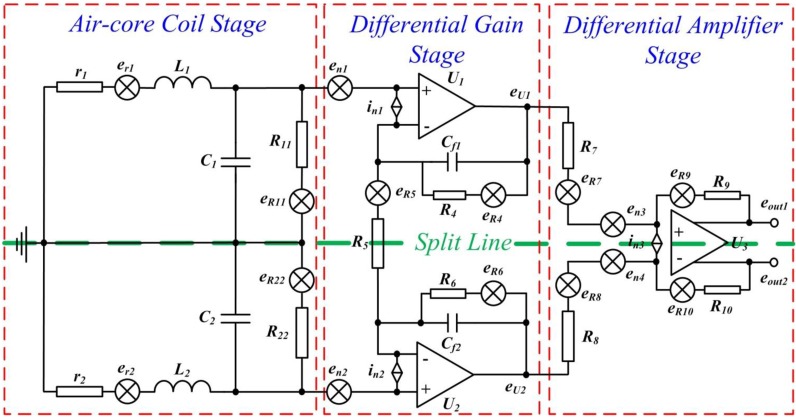
ACS circuit with equivalent noise locations.

The common-mode and parasitic signals observed in connecting wires are generally significantly reduced by using a differential structure. Figure 3 presents the schematic diagram of an original differential ACS and a differential air-core coil, integrated with a differential pre-amplifier, with noise locations.

The preceding figure illustrates that the ACS is symmetrical (top and bottom sections) as split by a green line in half. The ACS consists of three stages, namely, the air-core coil, differential gain, and differential amplifier stages. The air-core coil is used to effectively increase the magnetic flux density through the coil, which is indicated by the variations of the magnetic field. A two-stage differential amplifier is designed by using three operational amplifiers at the differential gain and differential amplifier stages to achieve a high gain and a high common-mode rejection ratio. Two resistors, *R_11_* and *R_22_*, are connected to the air-core coil in parallel as a matched resistor to adjust the working state of the air-core coil in accordance with the requirement of helicopter TEM exploration. Easy gain control is realized by changing the value of one single resistor *R_5_*, which can amplify the low amplitude sensed signal in the order of few mV to the operational range for further processing in several volts.

Noise contribution is the symmetrical structure of a pre-amplifier that can be analyzed in half. The EIN of ACS *V_en_* consists of three components (*i.e.*, input voltage noise *V_n_*, input current noise *I_n_*, and Nyquist noise $T_{n}=\sqrt{4kTR_{tot}}$ of all resistors in Figure 3) [11,12]. All these noise contributions can be combined to obtain *V_en_* as expressed below.

(6) $$V_{en}=\sqrt{V_{n}^{2}+{\left(I_{n}Z_{i,tot}\right)}^{2}+{\left(4kTR_{t,tot}\right)}^{2}}$$

where *Z_i,tot_* is the impedance of all resistors that input current noise flow through, and *T* denotes the environmental temperature at all resistance *R_t,tot_*.

*V_n_* from amplifiers *U_1_* and *U_3_* can be obtained as

(7) $$V_{n}=\sqrt{{\left(e_{n1}\right)}^{2}+{\left(\frac{e_{n3}}{G}\right)}^{2}}$$

where *e_n1_* and *e_n3_* are the input voltage noise of amplifiers *U_1_* and *U_3_*, respectively, and *G* is the gain of amplifier *U_1_*.

The noise *I_n_Z_i,tot_* generated by the input current noise of amplifiers *U_1_* and *U_3_* flowing through the three stages of ACS can be expressed as

(8) $$I_{n}Z_{i,tot}=\sqrt{{\left(i_{n1}Z\right)}^{2}+{\left(i_{n1}R_{eq1}\right)}^{2}+{\left(\frac{i_{n3}R_{eq2}}{G}\right)}^{2}}$$

where *i_n1_* and *i_n3_* are the input current noise of amplifiers *U_1_* and *U_3_*, respectively.

(9) $$Z=\left(r_{1}+j\omega L_{1}\right)||\frac{1}{j\omega C_{1}}||R_{11}=\frac{r_{1}+j\omega L_{1}}{1-\omega^{2}L_{1}C_{1}+\frac{r_{1}}{R_{11}}+j\omega (C_{1}r_{1}+\frac{L_{1}}{R_{11}})}$$

(10) $$R_{eq1}=R_{4}||\frac{R_{5}}{2}$$

(11) $$R_{eq2}=R_{7}||R_{9}$$

The Nyquist noise *T_n_* produced by all resistors included in Figure 3 can be obtained as

(12) $$\begin{array}{l} T_{n}=\sqrt{{\left(\frac{\sqrt{4kTr}}{r+j\omega L_{1}}Z\right)}^{2}+{\left(\frac{\sqrt{4kTR_{11}}}{R_{11}}Z\right)}^{2}+{\left(\sqrt{4kTR_{eq1}}\right)}^{2}+{\left(\frac{\sqrt{4kTR_{eq2}}}{G}\right)}^{2}}\\ \;=\sqrt{4kT\left(r\frac{Z^{2}}{{\left(r+j\omega L_{1}\right)}^{2}}+\frac{Z^{2}}{R_{11}}+R_{eq1}+\frac{R_{eq2}}{G^{2}}\right)} \end{array}$$

where ω is the angular frequency, ω = 2πf.

The total EIN *V_t,en_* can be calculated as $V_{t,en}=\sqrt{2}\cdot V_{en}$ depending on the complete symmetrical structure of the pre-amplifier.

The above analysis verifies the electrical parameters of the air-core coil in accordance with the requirement of helicopter TEM exploration. An ACS with low input noise can be achieved by adopting an operational amplifier with reasonable input voltage noise and input current noise. Selecting a resistor with an appropriate resistance is another means to obtain such an ACS. The optimization process of the specifications of the ACS is discussed in Section 4.

## 4. Specification Optimization of the ACS

### 4.1. Geometry Optimization of the Air-Core Coil

The geometry of the air-core coil is optimized to determine the values of *n* and *D* that meet the physical requirement of helicopter TEM exploration for a given air-core coil frame and a minimum acceptable wire diameter. However, this study does not optimize the air-core coil parameters as fixed factors. Moreover, the simulations are conducted solely on the basis of analytical Equations (2) and (5) to identify the best air-core coil geometry configuration.

**Figure 4:**
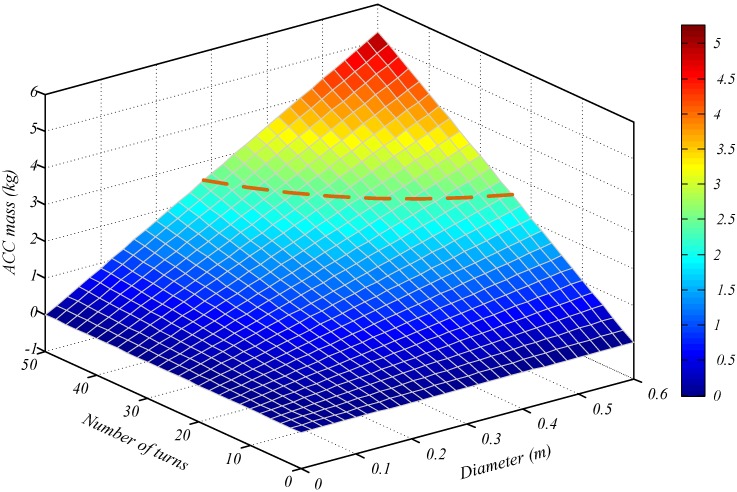
Contour map of the air-core coil mass *M* *versus* its *n* and *D.*

By using Equation (2), the contour map of the air-core coil mass *M* is shown in Figure 4, in which the value of *M* depends on those of *n* and *D*.

The orange line in the above figure represents the air-core coil mass *M* set as 2.5 kg. The aim for optimizing the geometry of the air-core coil is expressed as

(13) subject to M(n,D)=2.5 kg

**Figure 5:**
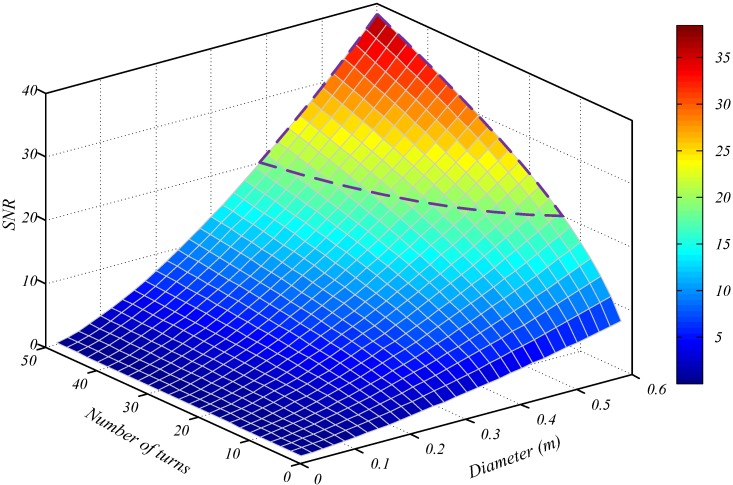
Contour map of the air-core coil SNR *versus* its *n* and *D*.

Figure 5 depicts the contour map of the air-core coil SNR, which is influenced by *D* and *n*, based on Equation (5).

The earth electrical conductivity in half-space models is generally set as 0.01 S/m. The deviation of detection depth caused by the ACS self-noise should not exceed 2%. As such, the air-core coil SNR should be at least 20 when the magnitude of the varying magnetic field (secondary field) is 1 nT/s. Accordingly, the optimum values of *D* and *n* can be calculated with the following equation:

(14) subject to SNR(n,D) ≥ 20

Figure 5 illustrates that the values of *D* and *n* in the defined zone (the area surrounded by the purple line) can satisfy the minimum required value of the air-core coil SNR.

**Figure 6:**
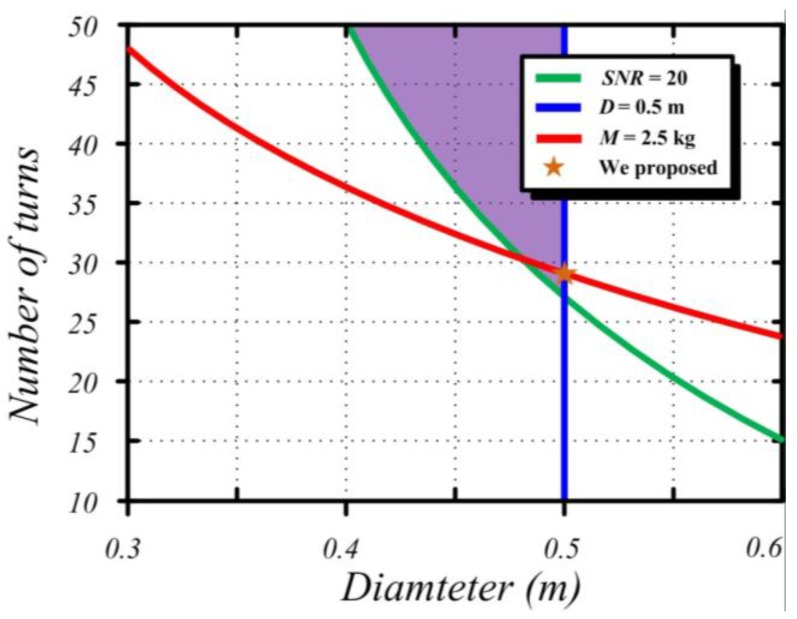
Contour maps of air-core coil mass *M*, size, and *SNR*.

To intuitively optimize the values of *D* and *n*, the contour maps of air-core coil mass *M*, size, and SNR are drawn in Figure 6.

In the above figure, the air-core coil mass *M* is set as 2.5 kg (as depicted by the red line) to reach the maximum load capacity in helicopter TEM exploration. The optimum value of *D* and *n* should be in line with the red line. The blue line shows that the diameter of the air-core coil is 0.5 m, which is the maximum dimension capacity of a helicopter TEM system. In practical applications, the diameter of air-core coil should be less than 0.5 m; hence, only the left part of the blue line in Figure 6 is accepted. Finally, the minimum SNR value of the air-core coil is configured as 20 (denoted as the green line) to guarantee that the deviation of detection depth would not exceed 2%. The SNR of the air-core coil in the upper part of the green line is higher than 20. On the basis of the above analysis, the optimum values of *D* and *n* are expressed as follows:

(15) $$\left\{ \begin{array}{l} subject\;to\;M(n,D)=2.5\text{ kg}\\ subject\;to\;D\;\leq \;0.5\text{ m}\\ subject\;to\;SNR(n,D)\;\geq \;20 \end{array}\right.$$

Correspondingly, the values of *D* and *n* in the red line as well in the purple shaded part all meet the stringent requirements for helicopter TEM exploration. The diameter is *D* = 0.5 m, and the number of turns is *n* = 28.

### 4.2. Optimization for Electrical Specification of the Pre-Amplifier

**Figure 7:**
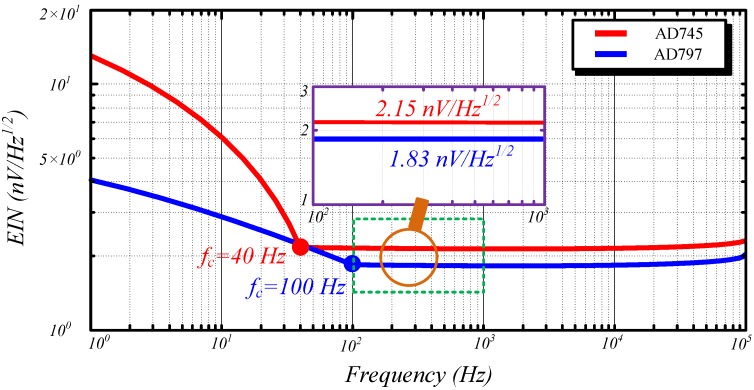
EINs of ACS calculated using AD797 and AD745.

To optimize the combination of the air-core coil and pre-amplifier, we investigated the EIN contributions of ACS by using different operational amplifiers. Two low noise operational amplifiers (*i.e.*, AD797 (Analog Devices Inc.) and AD745) are adopted. AD797 has an input voltage noise $V_{n}=0.9\text{ nV/}\sqrt{\text{Hz}}$, which is the lowest among the commercial operational amplifiers, and a relatively high input current noise $I_{n}=2.0\text{ pA/}\sqrt{\text{Hz }}@\text{ 2 kHz}$. Conversely, AD745 yields a slightly higher input voltage noise ${\text{V}}_{\text{n}}\text{=2.9 nV/}\sqrt{\text{Hz}}$ than AD797, and it has an extremely low input current noise ${\text{I}}_{\text{n}}\text{ = 6.9 fA/}\sqrt{\text{Hz }}@\text{ 2kHz}$. Figure 7 shows the EINs of ACS by using AD797 and AD745. The EINs consist of input voltage noise *V_n_*, input current noise *I_n_*, and Nyquist noise *T_n_*.

The whole frequency bandwidth can be divided into two regions (*i.e.*, *1/f* and broadband regions) [13,14]. In *1/f* region, EIN exhibits a *1/f* slope with regard to frequency and is reduced to its minimum value at the corner frequency. For AD745, the corner frequency is 40 Hz, which is smaller than that of AD797 (*i.e.*, 100 Hz). Frequencies higher than the corner frequency are called the broadband region, in which the EIN is flat and is the mainly usable bandwidth of helicopter TEM exploration. In this region, the EIN of AD797 and AD745 is as low as $\text{1.83 nV}\sqrt{\text{Hz }}$ and $2.\text{15 nV}\sqrt{\text{Hz }}$, respectively. Given that the distribution parameters of the designed air-core coil (e.g., resistance, capacitance, and inductance) are extremely small, their contributions to the EIN can be neglected at frequencies lower than 10 kHz. Nevertheless, the impedance of these parameters increases with increasing frequencies higher than 10 kHz. This case indicates that the magnitude of the EIN expands along with the increase of frequency. These finding simply that in using the designed air-core coil, AD797 produces a smaller EIN compared with AD745.

**Figure 8:**
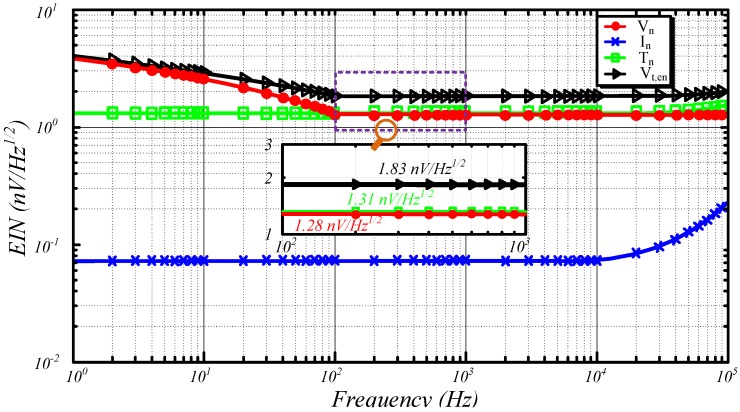
Noise source contribution map of ACS.

Considering that EIN consists of input voltage noise *V_n_*, input current noise *I_n_*, and Nyquist noise *T_n_*, the noise source contribution map is expressed as in Figure 8 to analyze the contributions of such noises to the EIN.

According to Figure 8, the *1/f* voltage noise is the main noise source when the frequency is lower than 100 Hz. When the frequency varies between 100 and 10 kHz, *V_n_* ($\text{1.28 nV/}\sqrt{\text{Hz }}$) and *T_n_* ($\text{1.31 nV/}\sqrt{\text{Hz }}$) both primarily generate the EIN. When the frequency increases to higher than 10 kHz, the impedance of the distribution parameters increases along with the EIN. Reducing *V_n_* and *T_n_* is helpful to minimize the EIN of the picked up ACS.

## 5. Experiment

### 5.1. Realization of ACS

**Figure 9:**
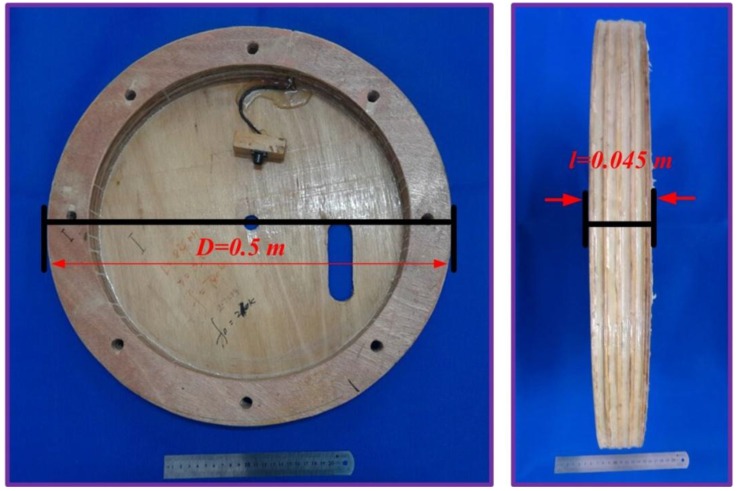
Experimental model of ACS.

Considering the optimum value of the geometric parameters from the model calculation, an experimental model of ACS was fabricated as shown in Figure 9.

The diameter *D*, number of turns *n*, and length *l* of the air-core coil are 0.5 m, 28, and 0.045 m, respectively. Furthermore, the resultant effective area of the air-core coil is 5.5 m^2^, and its mass is 2.5 kg with a ring wooden frame. By contrast, the length, resultant effective area, and mass of the 3D-3 are 0.6 m, 50 m^2^, and 16 kg, respectively. These data show that compared with the 3D-3, the designed ACS is more suitable for helicopter TEM exploration.

To detect the induced voltage, a low-noise operational amplifier AD797 was used to design and construct an appropriate electronic circuit in accordance with Figure 3. The specifications of the ACS are listed in Table 1.

**Table 1:** Fabricated parameters of ACS.

Parameters	Symbol	Value
Diameter of air-core coil	*D*	0.5 m
Diameter of wire	*d*	0.5 mm
Number of turns	*n*	28
Wooden frame density	ρ	730 kg/m^3^
Resistivity of wire	ρ_r_	1.7 × 10^−8^ Ω/m
Length of air-core coil	*l*	0.045 m
Resistance of air-core coil	*r_1_*	3.23 Ω
Inductance of air-core coil	*L_1_*	243.7 μH
Capacitance of air-core coil	*C_1_*	113 pF
Resistor	*R_4_* = *R_6_*; *R_5_*; *R_7_* = *R_8_*; *R_9_* = *R_10_*; *R_11_* = *R_22_*	1.5 kΩ; 100 Ω; 22.6 kΩ; 1 kΩ; 1.3 kΩ
Gain of pre-amplifier	*G*	672
Operational amplifier	*U_1_*, *U_2_*, *U_3_*	AD797

### 5.2. Frequency Response Comparison of ACS and 3D-3 Sensor

All measurements are performed inside a magnetic shielding room built with high permeability and inductivity materials. Correspondingly, this room provides the experiment with a sufficient shielding factor (*i.e.*, 40 dB) from both electric and magnetic field noises.

**Figure 10:**
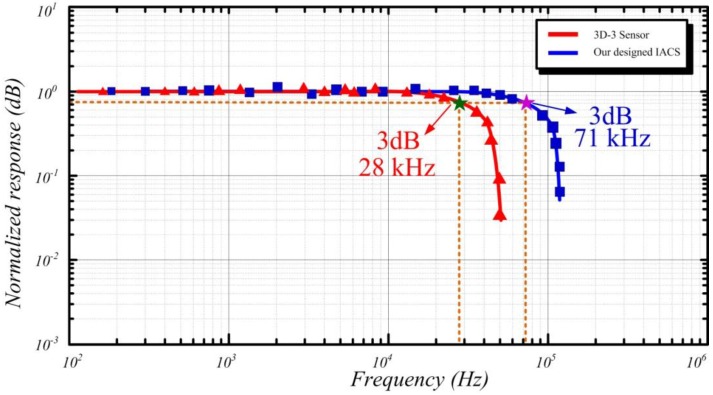
Comparison of the frequency response of the proposed sensors.

The frequency responses of the proposed sensors are first compared using a calibrated solenoid with a 13 nT/mA scale factor, which applies an external excitation field, to verify their respective frequency response performances. The calibrated solenoid is then driven by a current source generator (6221, Keithley Instruments Inc., Cleveland, OH, USA), and the induced output voltage of the sensors is measured using a dynamic signal analyzer (35670A, Agilent Technologies Inc. (Santa Clara, CA, USA)). Figure 10 shows the frequency response comparison of the fabricated ACS and 3D-3 sensor.

As shown in the preceding figure, the ACS works in a less under-damping state to guarantee that the received signal is free of distortion. The normalized response amplitude is flat until the frequency is higher than the self-resonant frequency at 71 kHz. After the self-resonant frequency, the response amplitude of ACS is rapidly decreased. Given that the resultant effective area of the commercial sensor 3D-3 is 50 m^2^, which is larger than that of the ACS, the 3 dB bandwidth can only go up to 28 kHz. Hence, the 3 dB bandwidth of the designed ACS is as high as 71 kHz, which meets the requirement of helicopter TEM exploration, with enough gain stability. The 3D-3 sensor is suitable for low frequency detection applications.

### 5.3. EIN of ACS

**Figure 11:**
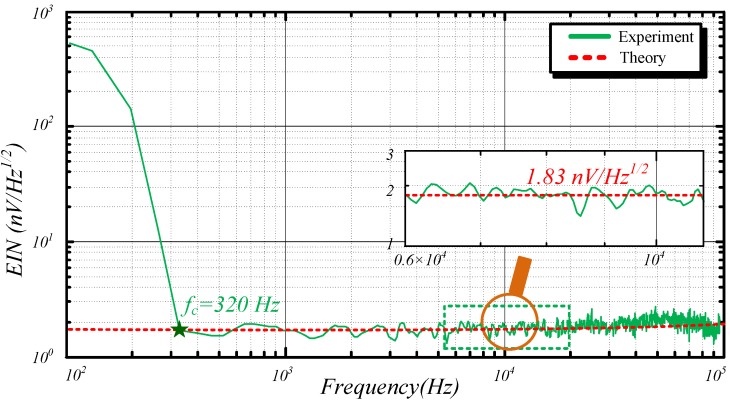
EIN of ACS.

The ACS is then placed in the center of the shielding room to achieve the greatest shielding factor, and the dynamic signal analyzer 35670A is employed outside the room to measure the EIN of ACS. Figure 11 displays the results.

The EIN in the *1/f* region linearly decreases with the increase of frequency until it reaches the corner frequency. Contrarily, the EIN in the broadband region is as low as $1.\text{83 nV}\sqrt{\text{Hz }}$ and can become 10 kHz. The EIN in this region is low compared with the geomagnetic field in the same frequency band. In addition, the EIN begins to rise with the growth of frequency higher than 10 kHz. The agreement between the experimental and simulation results shows the reliability of the ACS model with noise location. The self-corner frequency of the dynamic signal analyzer 35670A is 320 Hz, which is higher than the corner frequency of the designed ACS. As such, the testing result cannot exactly show the designed ACS specifications at frequency lower than 320 Hz.

Given that the ACS measures the time-varying magnetic field, its electrical performance is evaluated using the normalized value nV/m^2^. The normalized value equivalent to the square root of the integration of EIN^2^ in 3 dB bandwidth is described below.

(16) $$EIN_{nor}=\frac{\sqrt{\int_{B}EIN^{2}}}{S}$$

where *B* and *S* are the 3 dB bandwidth (71 kHz) and resultant effective area of the ACS (5.5 m^2^), respectively.

Consequently, the normalized EIN is calculated as 5.43 nV/m^2^.

### 5.4. Field Experiment

**Figure 12:**
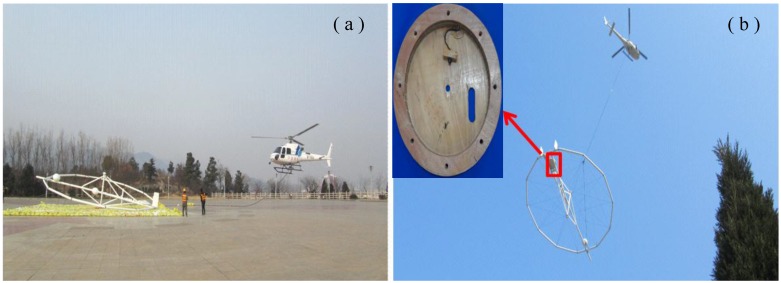
(**a**) Location site of the helicopter TEM system; (**b**) example of a helicopter TEM.

The research on mineral resources in Tongbai county in Henan province, China, has clearly improved in recent years. In this study, a helicopter TEM system is utilized to accelerate the rate of exploration in this area and to explore its geological features and metallogenic belt. In particular, large-scale helicopter TEM exploration is performed by using the designed ACS, which consists of a helicopter TEM system, to conduct a field experiment in this region. Figure 12 illustrates an example of a helicopter TEM system.

**Figure 13:**
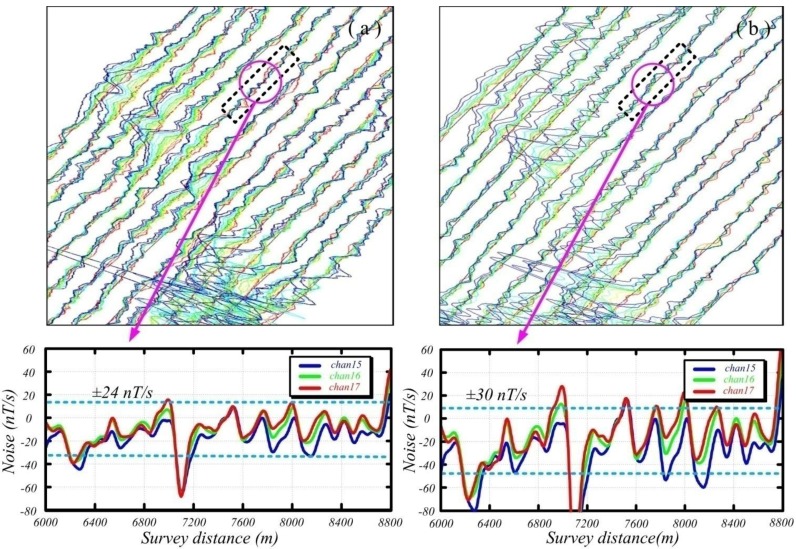
Cross-section maps of different survey lines detected by the (**a**) ACS-based helicopter TEM system and (**b**) the AeroTEM system.

In Figure 12b, the ACS is located in the bias center of the transmitting coil, which is shown in the red box. Correspondingly, the field experiment for the ACS-based helicopter TEM system and AeroTEM is compared, and the cross-section maps of different survey lines are shown in Figure 13.

The above figure illustrates that the anomaly shape and its location detected by the ACS-based helicopter TEM system and AeroTEM system are nearly the same. The last three sampling channels (*i.e.*, channels 15, 16, and 17) of these helicopter TEM systems are shown in the lower part of Figure 13, given that the cross-sections of these channels can reflect the performance of system noise. The noise of the helicopter TEM systems can reach ±24 and ±30 nT/s, respectively. This result shows the satisfactory performance of the designed ACS in accurately measuring the time-varying magnetic field of a helicopter TEM system.

## 6. Conclusions and Prospects

For this study, an ACS with a differential structure was designed, built, and tested to reduce the common-mode noise induced in helicopter TEM exploration surveys. The basic working principle and physical structure of the air-core coil with a ring wooden frame is elaborated, and a generalized electrical model of it is introduced. The schematic diagram of an ACS with noise location is introduced and analyzed correspondingly. To overcome the stringent performance requirements for helicopter TEM exploration, the design and optimization procedures are theoretically described and simulated in Matlab. The diameter and number of turns of the air-core coil as well as the operational amplifier of the pre-amplifier were designed and optimized. Moreover, an example of the ACS was built and tested. The mass, resultant effective area, 3 dB bandwidth, SNR, and normalized EIN were determined to be 2.5 kg, 5.5 m^2^ (diameter is 0.5 m), 71 kHz, 20 (exciting field strength is 1 nT/s), and 5.43 nV/m^2^, respectively. The conformity between the experimental and simulation results confirms the optimization theory. Finally, a field experiment was performed with a fabricated sensor to show the reliability of a helicopter TEM system based on the designed ACS.

Compared with other receiving sensors, the proposed ACS can have a smaller size, higher 3 dB bandwidth, and lower input noise. All these properties are highly critical for helicopter TEM exploration, particularly for its stringent performance requirements. Accordingly, the specifications of the ACS can be changed in accordance with the requirements of helicopter TEM exploration on the basis of the described optimization procedures. In our future works, the noise sources of the ACS (e.g., noise produced by attitude variations) for aerial flight will be explored.
